# The Transformative Potential of Liquid Biopsies and Circulating Tumor DNA (ctDNA) in Modern Oncology

**DOI:** 10.3390/diagnostics16040523

**Published:** 2026-02-09

**Authors:** Keren Rouvinov, Rashad Naamneh, Alexander Yakobson, Wenad Najjar, Mahmoud Abu Amna, Arina Soklakova, Ez El Din Abu Zeid, Ronen Brenner, Mohnnad Asla, Fahmi Abu Ghalion, Ali Abu Juma’a, Amichay Meirovitz, Walid Shalata

**Affiliations:** 1 The Legacy Heritage Cancer Center, Dr. Larry Norton Institute, Soroka Medical Center, Beer-Sheva 84105, Israelamichay@clalit.org.il (A.M.); 2 Goldman Medical School, Faculty of Health Sciences, Ben-Gurion University of the Negev, Beer-Sheva 84105, Israel; 3 Neurosurgery Department, Soroka Medical Center, Beer-Sheva 84105, Israel; 4 Neurosurgery Department, Hadassah Hebrew University Medical Center, Jerusalem 91200, Israel; 5 Department of Oncology, The Emek Medical Centre, Afula 18341, Israel; 6 Medical School for International Health, Faculty of Health Sciences, Ben Gurion University of the Negev, Beer Sheva 84105, Israel; 7 Edith Wolfson Medical Center, Oncology Institute, Holon 58220, Israel

**Keywords:** liquid biopsy, circulating tumor DNA (ctDNA), minimal residual disease (MRD), precision oncology, cancer

## Abstract

**Background**: Liquid biopsy, particularly through the analysis of circulating tumor DNA (ctDNA), represents a significant advancement in oncology. Unlike traditional tissue biopsies, ctDNA offers a minimally invasive, real-time approach to cancer management. It has demonstrated considerable potential in early cancer detection, monitoring of therapeutic responses, and assessing minimal residual disease (MRD) to predict recurrence. By enabling comprehensive molecular profiling through a simple blood test, ctDNA supports the core principles of precision oncology, facilitating more personalized and adaptive treatment strategies. **Methods**: In the following article we describe the recent developments focused on refining ctDNA detection assays to improve sensitivity and specificity. Advanced technologies, including next-generation sequencing (NGS) and digital PCR, are commonly employed. The integration of artificial intelligence (AI) and multi-omics approaches—such as combining genomic, epigenomic, and transcriptomic data—has further enhanced the analytical power of ctDNA assays. **Results**: Emerging evidence shows that ctDNA-based liquid biopsy enables dynamic, real-time tracking of tumor evolution and therapeutic resistance. Clinical studies have demonstrated its efficacy in detecting early-stage cancers, guiding treatment selection, and predicting relapse with higher accuracy than some conventional methods. Moreover, AI-enhanced algorithms have improved signal detection, allowing for more precise and earlier identification of actionable mutations and MRD. **Conclusions**: ctDNA analysis via liquid biopsy is poised to revolutionize cancer care by offering a non-invasive, precise, and adaptive tool for tumor characterization and monitoring. Although obstacles remain—particularly regarding assay sensitivity, standardization, and economic feasibility—ongoing technological innovations and multi-omics integration are rapidly advancing its clinical viability. With continued progress, ctDNA-based liquid biopsy is likely to become a cornerstone of routine oncology practice.

## 1. Introduction

**Figure 1:**
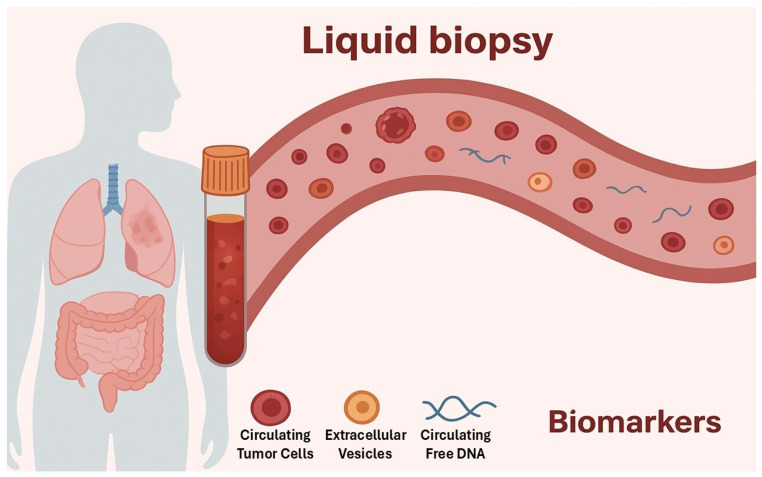
The figure illustrates the process and the main biomarkers detectable in a blood sample.

Liquid biopsy is an advanced molecular diagnostic approach that enables the analysis of tumor-derived components circulating in body fluids, most commonly blood. In addition to blood, other biofluids such as urine, saliva, and cerebrospinal fluid have also shown potential as valuable sources of biomarkers [1,2]. The main analytes assessed in liquid biopsy include circulating tumor DNA (ctDNA), circulating tumor cells (CTCs), extracellular vesicles (EVs), tumor-associated microRNAs, cell-free RNAs (cfRNAs), and tumor-educated platelets (Figure 1) [3]. Each offers distinct insights into tumor biology, progression, and treatment response.

Circulating Tumor Cells: Intact cancer cells shed from the primary tumor or metastases into the bloodstream. Their presence and quantity provide insights into tumor burden and progression. Extracellular Vesicles: Small particles released by tumor cells that carry proteins, RNA, and DNA fragments, reflecting tumor biology and signaling pathways. Circulating Free DNA: DNA fragments released from dying tumor cells. Analysis of cfDNA allows the detection of tumor-specific genomic alterations. MicroRNA: Small non-coding RNA molecules involved in gene regulation, often dysregulated in cancer, making them useful biomarkers for diagnosis and prognosis.

Unlike traditional tissue biopsy, which remains the gold standard for tumor diagnosis but requires invasive tissue sampling, liquid biopsy is minimally invasive and allows dynamic, real-time monitoring of cancer. Its advantages include the ability to detect specific tumor mutations across different stages of disease, evaluate treatment response, track minimal residual disease, and identify early recurrence. Importantly, it can be performed even in cases where surgical sampling is not feasible, such as in patients with difficult tumor localization or widespread metastatic disease [1,2,3].

**Figure 2:**
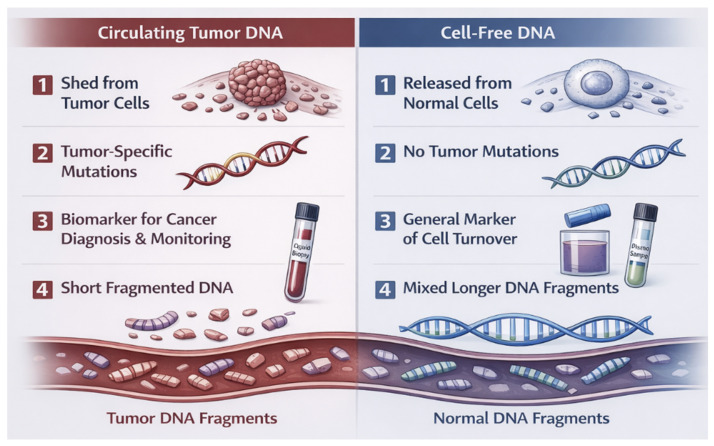
Comparison between ctDNA and cfDNA.

A critical distinction lies between cell-free DNA (cfDNA) and ctDNA. CfDNA represents a heterogeneous pool of DNA fragments released into the bloodstream through normal physiological processes, diet, infections, transfusions, or stress. In healthy individuals, cfDNA is present at very low levels (<10 ng/mL) and is typically ~167 base pairs in size. By contrast, ctDNA is a subset of cfDNA derived specifically from tumor cells, carrying genetic alterations identical to those of the primary tumor (Figure 2) [1,4,5]. While cfDNA is ubiquitous, ctDNA is virtually absent in healthy individuals, making its detection a highly specific marker for malignancy.

The discovery of cfDNA dates back to 1948, with its elevation in cancer patients first reported in 1977 [6]. For decades, however, oncology relied almost exclusively on tissue biopsies for diagnosis and molecular characterization. While essential for histological assessment, tissue biopsies are invasive, pose risks, and often fail to capture the spatial and temporal heterogeneity of tumors. Moreover, they are unsuitable for frequent monitoring. The introduction of liquid biopsy, particularly ctDNA analysis, represents a paradigm shift, offering repeated, non-invasive assessments that capture the evolving molecular landscape of cancer in real time [5,7].

Today, ctDNA has become a cornerstone of precision oncology, with applications in early detection, diagnosis, treatment monitoring, and personalized therapy selection. Its ability to mirror tumor genomic changes—including the emergence of resistance mutations or disease progression—provides clinicians with actionable information that enhances decision-making and patient outcomes.

## 2. Materials and Methods

### 2.1. Search Strategy

**Figure 3:**
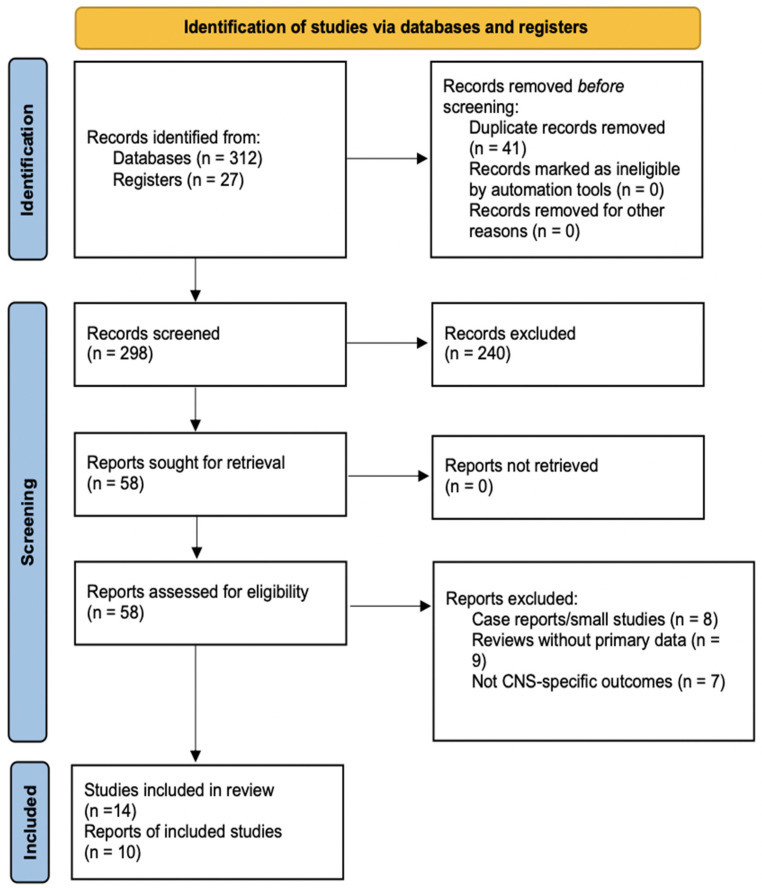
Flow diagram of the literature search.

A comprehensive literature search was conducted using the PubMed database to identify relevant studies published up to August 2025. The search incorporated a combination of keywords and Medical Subject Headings (MeSH) terms, including “Liquid Biopsy,” “Circulating Tumor DNA (ctDNA),” “Cancer Biopsy,” and “Minimal Residual Disease (MRD).” Boolean operators were employed to optimize and refine the search, ensuring a thorough retrieval of pertinent literature. The literature search flow, including study identification, screening, and selection, is presented in a PRISMA flow diagram (Figure 3).

Published meta-analyses included in this review were identified through the structured literature search and selected based on relevance to ctDNA clinical application, cancer type, methodological quality, and recency.

In addition to the literature review, three anonymized real-world cases of advanced lung adenocarcinoma from our institution were included to illustrate the clinical utility of ctDNA-based liquid biopsy in practice.

### 2.2. Inclusion Criteria

Studies were included if they provided relevant information, explanations, or updates related to liquid biopsy, including its applications and advancements in clinical or research settings.

### 2.3. Exclusion Criteria

Case reports, research or review articles with a limited number of patients or studies that mention liquid biopsy but do not report relevant outcomes (e.g., no data on ctDNA, MRD, sensitivity/specificity), studies lacking clear descriptions of how liquid biopsy was performed or analyzed, and studies that do not correlate liquid biopsy findings with clinical endpoints, such as treatment response, progression, or survival were excluded from the analysis.

## 3. Results

### 3.1. Biological Basis and Mechanisms of ctDNA Release

The presence of cfDNA in the bloodstream is a normal physiological phenomenon, originating from cellular byproducts released during routine processes, exogenous DNA from dietary intake or blood transfusions, or even from the nervous system in response to factors like stress [8]. The primary contributors to the overall cfDNA pool are cells from the hematopoietic system, accounting for approximately 55% of white blood cells and 30% from erythrocyte progenitors. ctDNA is a specific and highly informative subset of this cfDNA, comprising DNA fragments that are directly shed from tumor tissue and reflect the genetic alterations characteristic of the cancer [6].

The release of ctDNA into circulation occurs through a variety of distinct biological mechanisms, each contributing to the overall pool of tumor-derived fragments. A major pathway involves various forms of cell death, including apoptosis, necrosis, oncosis, ferroptosis, pyroptosis, and phagocytosis [9]. Apoptosis, a programmed form of cell death, typically releases shorter DNA fragments, often less than 200 base pairs (bp) in length, which are notably enriched with tumor-derived genomic alterations. These fragments are frequently packaged within apoptotic blebs, subsequently phagocytized by macrophages, and then released into the blood and lymphatic circulation. Conversely, necrosis, a more uncontrolled form of cell death, tends to yield larger DNA fragments, exceeding 200 bp, and is particularly prominent in advanced stages of cancer. Beyond cell death, living tumor cells can actively secrete ctDNA, often encapsulating these fragments within extracellular vesicles (EVs), which serve as protective carriers against degradation by circulating enzymes. Research indicates that nanoscale EV-derived DNA can offer superior mutation detection, particularly in early-stage non-small cell lung cancer (NSCLC). Other non-death-associated mechanisms, such as senescence and mitochondrial DNA (mtDNA) egestion, also contribute to the shedding of ctDNA [4,6].

CtDNA fragments possess distinct characteristics that aid in their identification and provide insights into their origin. They are typically small, ranging from 40 to 200 bp, with a prominent peak around 166 bp, corresponding to nucleosome-associated DNA fragments. Notably, ctDNA fragments are often shorter, averaging around 143 bp, compared to non-malignant cfDNA fragments, which are typically around 167 bp. The analysis of features such as fragment length, nucleosome occupancy, and preferred end motifs can provide valuable clues about the specific shedding mechanisms and help differentiate tumor-derived fragments from those originating from healthy cells. Fragments shorter than 100 bp, for instance, may be particularly enriched with tumor-derived genomic alterations [6].

The amount of ctDNA detectable in circulation is highly variable and influenced by a complex interplay of biological factors. Tumor type and overall tumor load play a significant role, with detection rates varying considerably across different malignancies. For instance, tumors in the central nervous system, kidneys, or prostate generally release lower levels of ctDNA, making detection more challenging. Conversely, ctDNA is more readily detectable in advanced stages of cancers such as ovarian, liver, pancreas, bladder, colon, lung, stomach, and breast cancers. A larger tumor bulk generally correlates with higher ctDNA levels. The anatomical location of the tumor also impacts ctDNA shedding, with tumors often releasing higher amounts of ctDNA into nearby body fluids, such as cystic fluid or peritoneal washings, than into the general bloodstream. Furthermore, tumors with high metabolic activity or those in advanced stages tend to release more ctDNA due to increased cellular turnover [10,11,12].

The dynamic equilibrium of ctDNA levels is also governed by clearance mechanisms. The half-life of cfDNA, including ctDNA, is remarkably short, typically ranging from 16 min to 2.5 h. This rapid clearance is mediated by circulating DNases, active clearance of nucleosomes, and filtration by organs such as the kidneys, liver, spleen, and lymph nodes. In cancer patients, excessive cell death can sometimes overload these natural clearance systems, leading to an accumulation of higher ctDNA levels in the circulation [6].

The diverse mechanisms of ctDNA release, including apoptosis, necrosis, and active secretion, coupled with the varying characteristics of the fragments (e.g., length, end motifs), indicate that ctDNA is not a singular biomarker but rather a complex collection of molecular signals. This suggests that future diagnostic platforms may need to analyze these nuanced features beyond simple quantification to enhance sensitivity and specificity, particularly for early detection, where ctDNA levels are inherently low. For instance, understanding whether ctDNA is primarily released via apoptosis versus necrosis could provide qualitative information about tumor activity and aggressiveness, moving beyond a simple “present/absent” or “how much” to a more refined “what kind” of ctDNA [13].

The remarkably short half-life of ctDNA, ranging from 16 min to 2.5 h, combined with its continuous shedding from tumor cells, means that ctDNA levels offer a true, real-time molecular snapshot of tumor activity. This dynamic nature represents a fundamental advantage over the static information provided by traditional tissue biopsies, enabling immediate feedback on treatment efficacy or disease progression. The rapid response of ctDNA levels to changes in tumor activity, such as increased cell death following therapy or accelerated growth, allows for immediate, actionable insights into treatment response or the emergence of resistance, often preceding any visible changes on conventional imaging. This real-time feedback loop is a core aspect of the revolution in cancer management, facilitating adaptive treatment plans and potentially preventing unnecessary toxicities or delayed interventions [14].

Furthermore, the ability of ctDNA to capture genetic alterations from multiple regions of a tumor, including both primary and metastatic sites, provides a more comprehensive representation of tumor heterogeneity than a single tissue biopsy. Tissue biopsies, by their nature, provide a localized snapshot of a single site. However, tumors are known to be genetically diverse, with different subpopulations of cells and metastatic lesions potentially harboring distinct mutations. By collecting DNA from various shedding tumor cells throughout the body, ctDNA offers a systemic, “liquid snapshot” of the entire tumor’s molecular landscape [9]. This capability is profoundly important for guiding targeted therapies and understanding resistance mechanisms, as ctDNA can reveal mutations that might not be present in the original biopsy or accessible metastatic sites, thereby directly influencing treatment selection and adaptation.

### 3.2. Technological Landscape for ctDNA Detection and Analysis

The effective analysis of ctDNA is critically dependent on the deployment of highly sensitive and specific molecular techniques, given its often-low abundance within the vast background of normal cell-free DNA in circulation.

#### 3.2.1. Overview of Highly Sensitive Detection Techniques

##### Next-Generation Sequencing (NGS)

Stands as a cornerstone technology in ctDNA analysis. This powerful method offers a significant advantage over traditional PCR-based approaches by enabling the detection of a remarkably broad range of genetic abnormalities [5,15]. NGS platforms are capable of comparing entire genes or even whole genomes to a reference sequence, allowing for the identification of previously unknown genetic changes across broad regions in a non-targeted manner. NGS has fundamentally transformed genomic research by facilitating the rapid and cost-effective sequencing of millions of DNA fragments simultaneously, a capability far exceeding that of older, single-sequence methods [16]. Specialized NGS platforms, such as the PacBio Onso system, are specifically engineered for ultra-low frequency mutation detection, capable of identifying tumor mutations at (VAFs as low as <1% or even <0.1%, which is crucial for the demanding requirements of ctDNA applications [17,18].

##### Polymerase Chain Reaction (PCR) Variants

Play a vital role in ctDNA detection, particularly for targeted analysis:

1. Quantitative PCR (qPCR) is utilized for the detection and quantification of specific, known mutations [5].
2. Digital PCR (dPCR) offers ultra-high sensitivity for the detection and absolute quantification of specific known mutations, often achieving a lower limit of detection than standard PCR or even some NGS approaches for highly targeted analyses. The majority of commercial kits for ctDNA detection are based on real-time PCR, which requires the design of specific primers that bind to defined sites in the DNA sequence.

The choice between NGS and PCR-based methods frequently involves a careful consideration of trade-offs. NGS provides broad, non-targeted detection of various genetic alterations, which is invaluable for comprehensive tumor profiling and the discovery of novel mutations. However, dPCR offers unparalleled sensitivity for known mutations, making it indispensable for detecting the very low levels of ctDNA characteristic of minimal residual disease (MRD). This suggests that a hybrid or sequential diagnostic approach, perhaps utilizing broad NGS for initial tumor profiling and then employing highly sensitive dPCR for targeted, longitudinal MRD monitoring, might represent the most effective and clinically impactful strategy. This is not merely a selection of technologies but a strategic application based on their inherent strengths and weaknesses, offering a more profound understanding of their clinical utility [5,19].

Beyond droplet-based platforms, chamber-based digital PCR (cdPCR) has emerged as an important technological advancement. cdPCR systems utilize microfluidic chips containing arrays of microchambers for the digital discretization of liquid samples. Following discretization, each microchamber functions as an independent reaction unit, enabling amplification and quantification of target molecules through fluorescence signal detection contrast to droplet-based dPCR, cdPCR improves analytical robustness by eliminating droplet fusion or rupture, increasing chamber independence, reducing cross-contamination risk, and enabling more stable and accurate quantification with improved chamber utilization. These features are particularly advantageous for applications requiring high analytical robustness, such as MRD detection [20].

#### 3.2.2. Types of Genetic Alterations Identified

The utility of ctDNA stems from its ability to carry specific genomic alterations that are characteristic of cancer cells, allowing for its differentiation from the more abundant normal cfDNA [21]. These detectable alterations include:

1. Point Mutations: Changes involving a single-nucleotide base within the DNA sequence.
2. Gene Amplifications: An increase in the number of copies of a specific gene.
3. Deletions: The removal of segments of genetic material.
4. Translocations: Rearrangements of genetic material, often between non-homologous chromosomes.
5. Epigenetic Changes: These include aberrant DNA methylation patterns, which are highly specific to tumor cells and can serve as crucial cancer biomarkers, particularly in scenarios where detectable mutations in ctDNA are absent.

While point mutations have traditionally been the primary focus of ctDNA analysis, the increasing emphasis on epigenetic changes, such as methylation patterns, and fragmentomics, which examines ctDNA fragment length and end motifs, signifies a progression towards a more sophisticated understanding of ctDNA. These non-mutational features provide additional layers of information that can significantly enhance the sensitivity and specificity of detection, especially in cases with a low mutational burden or for accurately identifying the tissue of origin [22]. The capacity for methylation to serve as a biomarker, particularly when mutations are not detectable, highlights an expansion of the diagnostic window for ctDNA beyond simple DNA sequence changes. This implies that multi-omics approaches are not merely about accumulating more data, but rather about capturing complementary biological signals that collectively improve diagnostic power, especially for difficult-to-detect early cancers.

#### 3.2.3. Assessment of Tumor Burden via Variant Allele Frequency (VAF)

The tumor burden can be quantitatively assessed by measuring VAF, a metric that quantifies the fraction of DNA sequencing reads containing a specific genetic variant or mutation within a given genetic locus. A higher VAF generally indicates a larger proportion of the DNA in the sample carrying the specific mutation, and a positive correlation between tumor volume and ctDNA VAF has been observed in various cancers, including NSCLC [19].

However, it is important to recognize that VAF serves as an indirect indicator of tumor size. A critical consideration is that a relatively small tumor exhibiting high cellular activity can release substantial amounts of ctDNA into the bloodstream, disproportionate to its physical size. This suggests that VAF is more accurately interpreted as a measure of tumor activity or cellular turnover rather than a static, direct measurement of tumor volume [19]. This nuanced distinction is crucial for interpreting treatment response, where a decrease in VAF might reflect reduced cellular proliferation or increased cell death induced by therapy, even in the absence of significant volumetric changes detectable by imaging. This refined understanding informs how clinicians interpret VAF kinetics for treatment monitoring, shifting the focus from a simple size measurement to a more dynamic assessment of the tumor’s biological response to intervention (Table 1).

### 3.3. Clinical Applications of ctDNA-Based Liquid Biopsy

The advent of ctDNA-based liquid biopsy has profoundly impacted cancer management across multiple clinical domains, offering a non-invasive, dynamic, and comprehensive approach to disease assessment. Its clinical applications extend from early cancer detection to monitoring therapeutic response, identifying MRD, and guiding personalized treatment strategies.

Early cancer detection represents one of the most promising uses of ctDNA-based assays. Liquid biopsy holds immense potential for identifying tumor-specific genetic changes at an earlier, often asymptomatic, phase of cancer development, which is paramount for improving clinical outcomes. This capability is particularly valuable for cancers that are typically diagnosed at late stages, such as pancreatic, lung, and ovarian cancers, where early detection can significantly alter prognosis [23].

In lung cancer, ctDNA and CTC analysis have demonstrated promising results for early detection. ctDNA levels have been shown to correlate with tumor volume and can be utilized to monitor the effectiveness of therapeutic interventions. Furthermore, CTC detection shows promise in identifying high-risk patients, such as smokers with chronic obstructive pulmonary disease (COPD), even before tumors become visibly apparent on imaging scans. A systematic review and meta-analysis specifically evaluating liquid biopsy for lung cancer diagnosis reported pooled sensitivity and specificity for ctDNA of 85% and 90%, respectively, underscoring its superiority as a biomarker for diagnosing early-stage lung cancer [24,25,26]. In colorectal cancer (CRC), CTC and cfDNA methylation profiling assays such as FDA-approved Epi proColon test have proven effective in detecting early-stage disease [27].

Similarly, in breast and pancreatic cancer, liquid biopsy can detect plasma cfDNA and exosomal microRNAs (miRNAs). Research indicates that profiling miRNAs in plasma can help differentiate early-stage breast cancer from healthy controls [28]. For pancreatic cancer, microRNA-based liquid biopsy assays have shown remarkable accuracy, achieving up to 97% for pancreatic ductal adenocarcinoma (PDAC) and 91% in earlier stages. The accuracy of these tests improves when combined with markers like CA 19-9 [29]. These findings mark a shift from passive early diagnosis to active cancer interception, envisioning a clinical future in which disease can be detected and managed before symptoms arise.

The emergence of Multi-Cancer Early Detection (MCED) assays, which are designed to integrate various molecular, immune, and metabolic markers from a single blood sample combined with artificial intelligence (AI) and metagenomic profiling further enhances diagnostic accuracy and facilitates personalized risk assessments [30].

Beyond early detection, liquid biopsy enables the dynamic evaluation of treatment response and disease progression. Serial measurements of ctDNA levels during treatment can offer early indications of response or resistance, often preceding any detectable changes through conventional radiographic imaging. The consistent association of ctDNA clearance or decrease with improved progression-free survival (PFS) and overall survival (OS) across multiple cancer types, such as NSCLC and malignant melanoma, elevates ctDNA kinetics beyond a simple diagnostic marker to a powerful prognostic and predictive biomarker of treatment efficacy [31].

For example, a meta-analysis of 32 studies involving 3047 NSCLC patients revealed that ctDNA decrease or clearance was significantly associated with improved PFS (pooled Hazard Ratio: 0.32, 95% CI: 0.26–0.40) and OS (HR: 0.31, 95% CI: 0.23–0.42) across various systemic therapies, including targeted therapy and immune checkpoint blockade (ICB) [32]. Similarly, in malignant melanoma, detectable ctDNA levels before or during ICB therapy were significantly associated with poorer OS (HR = 3.19 and 4.57, respectively) and PFS (HR = 2.08 and 3.79, respectively) [33].

Generally, ctDNA positivity or detection at any time point is linked to shorter PFS and OS, whereas ctDNA clearance or decrease during treatment is associated with a lower risk of progression and death [25]. This strong correlation suggests that ctDNA monitoring could become a primary endpoint in clinical trials and a key decision-making tool in routine practice, moving beyond simply detecting cancer to actively managing it based on molecular response. Furthermore, ctDNA may assist in distinguishing pseudoprogression—a temporary increase in tumor size due to immune cell infiltration—from true disease progression, which remains a significant challenge in interpreting responses to immunotherapy [19].

Another critical application is the detection of MRD. It refers to the persistence of a small number of tumor cells in the body after curative-intent treatment, which are often undetectable by standard radiological exams or clinical evaluation. The detection of MRD through liquid biopsy, particularly ctDNA, provides a valuable foundation for early prognosis assessment, recurrence monitoring, efficacy evaluation, and personalized treatment guidance. Postoperative ctDNA detection has been consistently associated with worse clinical outcomes and offers a crucial method to identify MRD before radiographic or clinical evidence of recurrence becomes apparent. The utility of ctDNA in detecting MRD post-treatment is critical for personalizing adjuvant therapy.

A positive MRD signal indicates a high risk of recurrence, which can guide decisions toward intensified treatment strategies. Conversely, a negative MRD signal might allow for de-escalation of treatment, potentially sparing patients from unnecessary toxicities and improving their quality of life. For instance, a randomized controlled trial in stage II colon cancer patients demonstrated that a ctDNA-guided approach resulted in a lower proportion of patients receiving postoperative adjuvant chemotherapy (15% vs. 28%) without compromising recurrence-free survival [27]. This directly addresses the unmet need of identifying which patients are truly cured after local therapies versus those who still harbor residual disease and require additional systemic treatment.

A significant advantage of ctDNA-based MRD detection is its ability to provide a substantial lead time before clinical diagnosis of recurrence. For example, in colorectal cancer, ctDNA positivity in postoperative plasma predicted cancer recurrence with 100% sensitivity and provided a median lead time of 9.4 months compared to carcinoembryonic antigen (CEA) elevation [27]. In a real-world multi-center study, ctDNA detection offered lead times before clinical diagnosis of up to 14.4 months for lung cancer, 19.5 months for colorectal cancer, 11.0 months for breast cancer, 5.5 months for gastric cancer, 6.8 months for liver cancer, and 8.9 months for ovarian cancer. Postoperative ctDNA positivity significantly increased the risk of recurrence, with Hazard Ratios exceeding 37 for lung, colorectal, breast, and gastric cancers [34]. This lead time allows for earlier intervention, which can be critical for improving patient outcomes.

Finally, liquid biopsy plays a pivotal role in guiding therapy selection by identifying actionable mutations, such as EGFR mutations in NSCLC, BRAF mutations in melanoma, or PIK3CA mutations in breast cancer [35]. This allows clinicians to select targeted therapies with higher precision, moving towards truly personalized medicine. Furthermore, liquid biopsy serves as a practical and effective alternative for molecular testing when traditional tissue biopsy is unfeasible due to tumor location, patient condition, or insufficient sample quality [36]. The dynamic nature of ctDNA also facilitates the real-time monitoring of tumor evolution and the early identification of acquired mutations that lead to drug resistance. A prime example is the detection of the T790M mutation in EGFR-mutated NSCLC, which signals resistance to first- and second-generation tyrosine kinase inhibitors, allowing for a rapid shift to alternative therapies like third-generation inhibitors (e.g., osimertinib) [37]. Unlike tissue biopsies, which provide a single spatial and temporal snapshot, liquid biopsy captures a more comprehensive, systemic view of the tumor’s molecular dynamics, offering crucial insights into tumor heterogeneity and clonal evolution. This comprehensive perspective is invaluable for optimizing real-time treatment strategies and overcoming therapeutic escape mechanisms (Table 2).

### 3.4. Advantages of Liquid Biopsy over Traditional Tissue Biopsy

Liquid biopsy offers several compelling advantages over traditional tissue biopsy, positioning it as a transformative tool in cancer management.

#### 3.4.1. Invasiveness and Patient Risk

The most significant advantage is its non-invasive and patient-friendly nature. Requiring only a simple blood draw, liquid biopsy minimizes patient discomfort, risks such as bleeding or infection, and the anxiety often associated with invasive surgical procedures [38]. This is particularly beneficial for individuals with advanced disease or those for whom surgical procedures are medically inadvisable.

#### 3.4.2. Real-Time Monitoring

Unlike tissue biopsies, which provide a static snapshot of the tumor at a single point in time, liquid biopsies offer repeatability for real-time monitoring. This allows for frequent, dynamic assessment of tumor evolution and response to treatment, providing invaluable insights into the ongoing biological processes within the patient [3]. In 2025, clinicogenomic study of NSCLC and SCLC undetectable ctDNA tumor-fraction levels after therapy initiation correlated with longer progression-free and overall survival (23.5 vs. 9.5 months in NSCLC, HR = 0.34; 15.9 vs. 8.3 months in SCLC, HR = 0.19), supporting ctDNA as a biomarker for real-time assessment of tumor response [39].

#### 3.4.3. Tumor Heterogeneity

Liquid biopsy excels at overcoming tumor heterogeneity, a fundamental challenge for tissue biopsies. Tissue biopsies typically sample a single, localized region of a tumor, which may not fully capture the spatial and temporal molecular variations present across the entire tumor or its various metastatic sites [40]. Liquid biopsy overcomes the limitations of single-lesion tissue sampling by capturing tumor heterogeneity and tracking clonal evolution across metastatic sites, enabling noninvasive detection of emerging resistance mutations and guiding therapy rechallenge—such as the ctDNA-based anti-EGFR retreatment strategies validated in the CHRONOS and CRICKET trials for RAS/BRAF wild-type metastatic colorectal cancer. ctDNA provides a more comprehensive, systemic view of the tumor’s molecular dynamics, reflecting genetic alterations from multiple regions and evolving clones [41]. This ability to capture the evolving molecular landscape of the tumor is crucial for adaptive treatment strategies.

#### 3.4.4. Sample Accessibility

For deep-seated or inaccessible tumors, where traditional tissue biopsy may be impractical or carry high surgical risks, liquid biopsy serves as a viable and effective alternative. It can also be utilized when tissue samples are of insufficient quality or quantity for molecular analysis, a common limitation in clinical practice [38].

#### 3.4.5. Turnaround Time

Finally, liquid biopsy generally offers faster turnaround times for results. Typically, results are available within 10 days, often quicker than the prolonged waiting periods associated with traditional tissue biopsy results [42]. This efficiency can facilitate faster initiation of first-line therapy, which is critical in rapidly progressing cancers.

While liquid biopsy offers significant advantages, it is important to understand that it currently complements rather than replaces traditional tissue biopsy. Tissue biopsy remains the gold standard for definitive initial diagnosis and detailed histological and cellular architectural insights. However, the dynamic, systemic, and non-invasive nature of liquid biopsy makes it indispensable for ongoing disease management, the detection of resistance mechanisms, and the assessment of minimal residual disease. This implies a future characterized by integrated diagnostics, where both methods are utilized synergistically to provide the most comprehensive and effective patient care.

The ability of liquid biopsy to overcome the “snapshot” limitation inherent in tissue biopsies fundamentally transforms how tumor evolution is understood and managed. Tissue biopsies provide a static, single-point-in-time sample, yet tumors are dynamic entities that continuously evolve and acquire new mutations, particularly under therapeutic pressure. ctDNA’s real-time monitoring capability means that clinicians can track these evolutionary changes, especially the emergence of resistance mechanisms, as they occur [42]. This dynamic molecular insight allows for truly adaptive treatment strategies, where therapy can be modified as soon as molecular resistance is detected, potentially prolonging treatment efficacy and improving patient outcomes (Table 3).

### 3.5. Current Challenges and Limitations in ctDNA Application

Despite the transformative potential of ctDNA-based liquid biopsies, their widespread clinical implementation faces several significant challenges and limitations. Addressing these hurdles is crucial for realizing the full promise of this technology.

#### 3.5.1. Assay Sensitivity and Specificity

A primary challenge is the limited detection capability of ctDNA, particularly in early-stage cancers where the tumor burden is low and ctDNA levels are often below the detection limits of current methods. This inherent biological constraint can lead to false-negative results, potentially missing early disease or residual cancer. Furthermore, even in advanced cancers, the sensitivity of ctDNA detection can vary considerably depending on the specific driver gene being analyzed. For instance, in advanced NSCLC, sensitivity ranged from 0.29 for ROS1 mutations to 0.77 for KRAS mutations [24].

Conversely, false-positive results can occur, predominantly due to clonal hematopoiesis (CH). CH involves the presence of somatic mutations in blood cells that are unrelated to the cancer, a common incidental finding, especially in older patients. Differentiating these CH-derived mutations from true tumor-derived alterations poses a significant challenge for accurate interpretation. Additionally, radio(chemo)therapy can induce variants in hematopoietic cells or exposed tissue, which can be difficult to distinguish from genuine tumor variants, particularly when they appear at low variant allele frequencies [43].

The persistent challenge of low sensitivity in early-stage cancers creates a critical gap between promising research findings and widespread clinical utility for population screening. While MCED tests are emerging, their broad societal impact is contingent on overcoming this sensitivity barrier to avoid high false-negative rates in healthy populations, which could lead to missed early interventions [44]. Without significant technological breakthroughs in ultra-sensitive detection, widespread population-level screening for early cancer detection remains challenging, as the risk of missing true early cancers could outweigh the benefits.

#### 3.5.2. Lack of Standardization and Reproducibility

Significant discrepancies in results often arise due to the diverse standards and interpretations employed by different ctDNA testing methods and laboratories. A critical need exists for the establishment of standardized testing protocols and interpretative guidelines to ensure consistency and reliability across various clinical settings [1]. Pre-analytical biases, including variations in blood drawing techniques, types of collection tubes, plasma separation methods, DNA extraction methodologies, sample conservation, and even the specific equipment used, can introduce substantial variability into the results, making reproducible assays difficult across different centers [45]. This pervasive lack of standardization across pre-analytical, analytical, and post-analytical phases represents a critical systemic barrier. Without robust, reproducible protocols, clinical utility cannot be definitively proven in large-scale trials, which subsequently hinders regulatory approval, consistent reimbursement, and broad physician confidence. This implies that collaborative efforts from industry, academia, and regulatory bodies are essential to establish universal guidelines, which are a foundational requirement for liquid biopsy to transition from promising research to routine clinical practice, impacting its economic and societal adoption.

#### 3.5.3. Cost Implications and Reimbursement Hurdles

The high cost associated with ctDNA detection technologies and equipment presents a significant barrier to their widespread application and comprehensive clinical monitoring. This financial burden can limit accessibility for routine use, particularly in resource-constrained healthcare settings. Furthermore, inconsistent or absent reimbursement policies exacerbate this challenge. For example, in Taiwan, the National Health Insurance (NHI) system currently reimburses EGFR mutations detected through tissue biopsy but not those identified via liquid biopsy, largely due to concerns about the lower concentration of ctDNA in blood and its implications for sensitivity in clinical settings [46].

#### 3.5.4. Need for Robust Randomized Clinical Trials

Despite the increasing volume of studies on liquid biopsy, there remains a lack of randomized controlled trials (RCTs) that definitively demonstrate its clinical benefit for applications such as MRD detection. While many studies have shown a satisfactory positive predictive value for ctDNA detection, indicating that its presence is associated with worse disease-free survival, the negative predictive value (NPV) of the technique often remains low. This means that an undetectable ctDNA signal after curative-intent treatment does not reliably predict the definitive elimination of the tumor, largely due to the technical limitations mentioned previously [44]. More carefully designed RCTs are needed to evaluate whether treatment intensification improves outcomes in MRD-positive patients and if treatment de-escalation is safe in MRD-negative patients. The call for rigorous clinical studies to prove clinical utility highlights that despite the excitement, a significant evidence gap remains. Widespread adoption before conclusive mortality benefit is proven could lead to unintended harms, including over-diagnosis and misallocation of resources. This emphasizes the critical role of randomized controlled trials as the “gold standard” to ensure responsible integration into clinical practice [44].

#### 3.5.5. Unclear Tissue of Origin

When ctDNA is detected in an asymptomatic individual, especially for common mutations like TP53, it can be unclear from which specific tissue the mutation originated. This ambiguity complicates the interpretation of results for general cancer screening. The challenge of an unclear tissue of origin and the confounding presence of clonal hematopoiesis in asymptomatic individuals highlight a profound ethical and clinical dilemma. Detecting a ctDNA signal without knowing its precise source or definitive clinical significance (e.g., whether it represents a nascent tumor or benign clonal hematopoiesis) raises serious questions about the appropriate clinical response, the potential for over-diagnosis, and the induction of patient anxiety, even before considering the question of cost [21]. This implies that simply detecting a signal is insufficient; there must be a clear, evidence-based pathway for actionability. Without this, widespread population screening could lead to unnecessary follow-up procedures, patient distress, and increased healthcare costs without clear, proven benefit.

#### 3.5.6. Other Limitations

Other limitations include the fact that current evidence does not suggest that ctDNA can fully replace traditional pathological testing. Additionally, insufficient cfDNA input during sample processing can impair results, and discordant calls often occur at low VAFs, (Table 4) [43].

### 3.6. Emerging Technologies and Future Directions

The future of liquid biopsy and ctDNA analysis is characterized by rapid technological advancements and increasing integration with complementary scientific disciplines, promising to overcome current limitations and expand clinical utility.

#### 3.6.1. Integration with Artificial Intelligence and Machine Learning

Artificial intelligence is profoundly transforming healthcare and is becoming an indispensable component for unlocking the full potential of liquid biopsy. AI and machine learning algorithms are uniquely positioned to process vast, heterogeneous datasets, including genomic, transcriptomic, proteomic, clinical trial outcomes, imaging, and demographic information, at unprecedented speed and scale. This capability enables a more holistic and nuanced understanding of each patient’s condition [47].

AI-driven platforms are revolutionizing biomarker discovery by efficiently sifting through large-scale datasets to identify novel biomarkers associated with specific cancer subtypes or therapeutic responses. These insights empower clinicians to stratify patients more precisely and select therapies with a higher probability of success based on individual molecular features. Furthermore, ML algorithms learn from processed data, continuously improving detection accuracy and reducing the incidence of misidentified cases. AI can integrate diverse molecular, immune, and metabolic data to generate personalized cancer risk profiles, tailoring prevention strategies to an individual’s unique biological signatures [48]. The integration of AI and ML is not merely an add-on; it is becoming the essential computational engine that unlocks the full potential of liquid biopsy. AI’s ability to process vast, complex multi-omics data and identify subtle patterns is critical for overcoming the data interpretation challenges that limit current ctDNA assays [46]. This implies that the future of liquid biopsy is inextricably linked to AI’s analytical power, moving from raw data to actionable clinical insights.

Moreover, AI enables continuous, real-time tracking of cancer development and treatment effects by monitoring biomarker changes in body fluids. This dynamic surveillance allows clinicians to adjust treatment plans without delay and to recognize subtle DNA changes that may indicate early cancer recurrence [49,50].

#### 3.6.2. Multi-Omics Approaches

An increasingly important direction involves the integrative analysis of different tumor-derived omics data, including genomics, epigenetics, fragmentomics, and proteomics, from various body fluids. This multi-omics approach has demonstrated superior performance compared to the analysis of single-modality data alone, as different omics layers offer complementary information for interrogating cancers.

Multi-omics significantly improves sensitivity and accuracy for early cancer detection and MRD monitoring, particularly in cases with very low tumor burden or ctDNA concentration. For example, studies combining multiple genomic features (e.g., 5-Hydroxymethylcytosine, nucleosome footprint, motif, and fragmentation) have achieved exceptionally high sensitivity and specificity (AUC > 0.99) for early detection of hepatocellular carcinoma (HCC). Similarly, integrated genomic strategies combining cfDNA mutations, background artifacts, fragment size, and CNVs have robustly detected early lung cancers [51]. The shift towards multi-omics integration signifies a recognition that a single biomarker, such as mutations, is often insufficient, especially for early detection or in the context of complex tumor heterogeneity. By combining diverse data types, a more holistic and robust “fingerprint” of the tumor can be obtained, significantly improving diagnostic accuracy and predictive power.

Multi-omics also enhances MCED and Tissue of Origin (TOO) localization. The CancerSEEK blood test, for instance, integrates cfDNA mutations and protein biomarkers to achieve high sensitivity and specificity for MCED and TOO localization across multiple cancer types. This approach can further be boosted by integrating clinical risk factors and imaging data. For improved MRD monitoring and treatment response surveillance, multi-omics strategies enhance assay performance even at low ctDNA concentrations. Combining ctDNA with CTCs has improved MRD detection for early-stage Triple-Negative Breast Cancer patients, enhancing the prediction of disease recurrence [51].

#### 3.6.3. Role in Accelerating Drug Development and Clinical Trial Design

ctDNA is increasingly recognized as a valuable biomarker for accelerating drug development and refining clinical trial design. Its utility lies in its ability to enrich high-risk populations for clinical trials, thereby maximizing therapy efficacy and potentially decreasing the necessary study size. This allows researchers to focus on patient cohorts most likely to benefit from additional treatment, potentially moving new therapies ahead of next-line treatments.

Furthermore, ctDNA can predict therapy response much sooner, often as early as 6 weeks into treatment [52,53]. This capability supports early therapy efficacy readouts, serving as a co-primary or surrogate endpoint in clinical trials. Such early insights can significantly improve program prioritization and planning within pharmaceutical companies and potentially accelerate the regulatory approval of new therapies. The FDA’s draft guidance on the use of ctDNA in early-stage solid tumor drug development underscores its potential to assist and expedite the drug development process. The FDA’s recognition of ctDNA as a biomarker for drug development represents a significant regulatory endorsement [46]. Its ability to enrich high-risk populations and provide early efficacy readouts means ctDNA can streamline clinical trials, potentially reducing costs and accelerating the availability of new cancer therapies.

#### 3.6.4. Novel Assay Development

Continuous advancements in sequencing technologies are crucial for improving the sensitivity of ctDNA detection. Novel assay developments, such as those utilizing the PacBio Onso system, are enabling the detection of tumor mutations at ultra-low frequencies, specifically less than 1% or even 0.1% [18]. This enhanced sensitivity is critical for expanding the applicability of ctDNA to earlier stages of cancer and for more precise MRD monitoring, where ctDNA is present in minute quantities. These technological leaps are fundamental to overcoming the current limitations in detection capability.

### 3.7. Societal, Economic, and Ethical Implications

The rapid evolution and adoption of liquid biopsy technologies, particularly those centered on ctDNA, carry significant societal, economic, and ethical implications that warrant careful consideration as they become more integrated into healthcare systems.

#### 3.7.1. Market Growth and Economic Impact

The global liquid biopsy market is projected for substantial growth, reflecting its increasing importance in cancer care. Forecasts indicate a rise from $4.6 billion in 2024 to an estimated $13.1 billion by the end of 2029, demonstrating a robust compound annual growth rate (CAGR) of 23.3%. The surge reflects liquid biopsy’s rising importance in cancer care, as stakeholders increasingly invest in and adopt blood-based diagnostics. This impressive growth is fueled by the rising global prevalence of cancer, continuous technological advancements (especially in NGS), and a growing demand for early, non-invasive diagnostic methods. North America presently dominates the market, a position attributed to its well-established healthcare infrastructure and favorable reimbursement policies that support the adoption of these advanced technologies [54]. While liquid biopsy offers potential economic advantages by reducing the need for repeat invasive tissue biopsies and their associated complications, its overall cost-effectiveness remains uncertain, and further dedicated health-economic studies are required to clarify its value in routine clinical practice.

#### 3.7.2. Potential for Widening Health Inequities and Access to Care

Despite the clinical promise of liquid biopsies, their high costs and patchy insurance coverage pose serious challenges to equitable access. A single ctDNA test can cost around $500–$3000, and in many healthcare systems patients must pay out-of-pocket due to lack of reimbursement. If these financial and policy barriers are not proactively addressed, the widespread adoption of MCED tests, particularly before conclusive evidence of mortality reduction, risks exacerbating existing health inequities in cancer screening and early diagnosis. The rapid market growth and transformative potential of liquid biopsy are juxtaposed with significant barriers to access, particularly cost and reimbursement. This implies that without proactive policy and regulatory frameworks, this revolutionary technology risks widening existing health inequities, potentially creating a two-tiered system of cancer care where only privileged populations can fully benefit [55].

#### 3.7.3. Ethical Considerations in Population Screening and Genetic Information

The integration of liquid biopsy into population-wide screening initiatives introduces several ethical considerations. While MCED tests are designed with high specificity, the potential for false positives and the subsequent need for extensive, often invasive, follow-up diagnostics can lead to considerable patient anxiety, unnecessary procedures, and substantial out-of-pocket expenses. The challenge of clonal hematopoiesis (CH), where benign mutations in blood cells can mimic tumor signals, further complicates interpretation and increases the risk of false positives [43].

Moreover, detecting a ctDNA signal in an asymptomatic individual without a clear understanding of its tissue of origin or a well-defined clinical action pathway raises critical questions about its utility and potential for harm. Simply detecting a signal is insufficient; there must be a clear, evidence-based pathway for actionability. Without this, widespread population screening could lead to unnecessary follow-up procedures, patient distress, and increased healthcare costs without clear benefit. Furthermore, liquid biopsy provides highly sensitive genetic information, necessitating robust ethical oversight and stringent data privacy measures to protect patient confidentiality and prevent misuse of genetic data [45].

Finally, the implementation of MCED screening must grapple with data privacy issues. Liquid biopsies generate vast amounts of personal genetic information. If widely deployed, they could largely create a genomic profile on large segments of population. This reality demands robust ethical oversight and strict data protection measures to safeguard patient confidentiality [56].

#### 3.7.4. Policy and Regulatory Challenges for Widespread Adoption

The rapid pace of evidence development and the inherent heterogeneity in data for different liquid biopsy applications (e.g., screening, treatment selection, monitoring) create uncertainty around technology performance for regulators, payers, and healthcare providers. This dynamic environment makes it challenging for stakeholders to remain updated on key developments and confidently assess the performance of liquid biopsies.

A substantial lack of familiarity and educational barriers exist for both healthcare providers and patients regarding the appropriate use cases, interpretation, and integration of liquid biopsy results with traditional diagnostics. This necessitates comprehensive educational initiatives to ensure informed decision-making [45].

Finally, the absence of widespread regulatory approval for many MCED tests and the complexity of coverage decisions significantly hinder widespread adoption. To facilitate broader clinical integration, there is a critical need for a comprehensive evidence package that clearly outlines the analytic validity (a test’s ability to accurately measure the biomarker), clinical validity (its ability to identify true diagnoses or differentiate patient groups with distinct outcomes), and clinical utility (its ability to improve patient outcomes) for each specific use case [57].

MCED tests, while promising, are “additive to, not replacements for, current cancer screening tests”. Their ability to detect multiple cancers simultaneously, often with an unknown tissue of origin, necessitates a re-evaluation of established screening paradigms and the development of entirely new clinical pathways for follow-up and management of positive results. A positive MCED result is not a diagnosis but a signal, requiring a complex diagnostic workup pathway that could lead to patient anxiety, over-investigation, and significant costs if not managed carefully. This represents a profound implication for healthcare systems and clinical practice, demanding careful planning and comprehensive education.

## 4. Discussion

To fully realize the transformative promise of liquid biopsy and ctDNA in oncology, concerted efforts across research, clinical practice, and policy are essential. The following recommendations are crucial for advancing their responsible and equitable integration into routine clinical care:

### 4.1. Standardization

It is imperative to prioritize the development and widespread adoption of standardized protocols for ctDNA sample collection, processing, and analysis across all clinical and research settings. This will ensure reproducibility and reliability of results, which are foundational for clinical confidence and regulatory acceptance.

### 4.2. Rigorous Clinical Validation

Continued investment in large-scale, prospective, randomized controlled trials is critical to definitively establish the clinical utility and cost-effectiveness of ctDNA-guided interventions. This is particularly important for applications such as early detection in asymptomatic populations and for guiding treatment de-escalation or intensification based on MRD status.

### 4.3. Enhanced Sensitivity and Specificity

Ongoing research and development efforts must focus on novel assay technologies, including ultra-sensitive sequencing platforms and multi-omics integration, to further improve the sensitivity of ctDNA detection, especially for early-stage cancers, while simultaneously mitigating false positives arising from clonal hematopoiesis.

### 4.4. Educational Initiatives

Comprehensive educational programs are needed for both healthcare providers and patients. These programs should foster a deep understanding of liquid biopsy’s benefits, inherent limitations, and its appropriate integration into existing clinical workflows, ensuring informed decision-making and optimal patient management.

### 4.5. Policy and Reimbursement Frameworks

Proactive development of clear and equitable policy and reimbursement guidelines is essential to support broad patient access to ctDNA testing. These frameworks must consider the evolving evidence base and actively address the significant cost barriers that currently limit widespread adoption.

### 4.6. Integrated Diagnostics

Promoting the synergistic use of liquid biopsy with established diagnostic modalities, such as traditional tissue biopsies and advanced imaging techniques, will provide the most comprehensive and dynamic picture of a patient’s cancer. This integrated approach leverages the strengths of each method for optimal patient care.

By systematically addressing these recommendations, the full potential of liquid biopsy and ctDNA can be realized, leading to more timely, personalized, and ultimately more effective cancer care for a wider population.

## 5. Conclusions

Liquid biopsies, propelled by remarkable advancements in ctDNA analysis, are fundamentally reshaping the landscape of cancer care. They offer a non-invasive, real-time, and comprehensive approach to disease management, proving invaluable for early cancer detection, dynamic monitoring of treatment response, and precise assessment of minimal residual disease. The strategic integration of artificial intelligence and sophisticated multi-omics approaches further amplifies their potential, promising to usher in an era of even more personalized and effective cancer strategies. While the potential is revolutionary, the proven clinical utility for all applications is still evolving and requires rigorous validation.

## Figures and Tables

**Table 1:** Key Methods for ctDNA Detection and Their Characteristics.

Method	Principle	Strengths	Weaknesses	Key Applications	Sensitivity/VAF
Next-Generation Sequencing (NGS)	Massively parallel sequencing of DNA fragments	- Broad genomic coverage	- Higher cost	- Comprehensive tumor profiling	Detects broad range of VAFs; sensitivity improves with deep sequencing (e.g., <0.1% VAF with PacBio Onso)
		- Non-targeted detection (SNVs, CNVs, fusions)	- Potentially lower sensitivity for ultra-low VAFs than dPCR	- Identifying actionable mutations	
		- Comprehensive profiling	- Longer turnaround time (rapid-NGS protocols are being developed to address this)	- Resistance mechanism detection	
		- Identifies novel mutations		- Multi-gene panels	
Digital PCR (dPCR)	Absolute quantification by partitioning sample into thousands of reactions	- Ultra-high sensitivity for known mutations	- Limited to known mutations	- MRD detection	Ultra-high sensitivity; detects VAFs as low as 0.002%
		- Absolute quantification	- Less comprehensive than NGS	- Monitoring resistance mutations	
		- High precision at low abundance	- Requires prior knowledge	- Highly sensitive detection of low VAFs	
Quantitative PCR (qPCR)	Amplification and quantification of specific DNA sequences	- Fast and cost-effective	- Lower sensitivity than dPCR	- Rapid detection of common driver mutations	Good for common mutations; limited sensitivity for very low VAFs
		- Good for specific, common mutations	- Limited to known mutations	- Initial screening for known alterations	
			- Less comprehensive than NGS		

**Table 2:** Summary of ctDNA performance across cancers based on published meta-analyses.

Application/Cancer Type	Metric	Value (95% Confidence Interval)	Source
Early Detection (Lung Cancer)	Sensitivity	85%	[26]
	Specificity	90%	[26]
Treatment Response Monitoring (NSCLC)	PFS HR (ctDNA decrease/clearance)	0.32 (0.26, 0.40)	[32]
	OS HR (ctDNA decrease/clearance)	0.31 (0.23, 0.42)	[32]
MRD Detection (Colorectal Cancer)	Sensitivity for recurrence prediction (post-op plasma)	100%	[27]
	Lead time to radiological recurrence vs. CEA elevation	9.4 months	[27]
	Post-op ctDNA positivity HR for recurrence	44.3 (11.3, 173.2)	[34]
Prognostication (Lung Cancer)	PFS HR (ctDNA positivity at any time point)	2.34 (1.89, 2.89)	[25]
	OS HR (ctDNA positivity at any time point)	2.33 (1.91, 2.85)	[25]
Prognostication (Malignant Melanoma)	OS HR (detectable ctDNA pre-ICI therapy)	3.19 (2.22, 4.58)	[33]
	PFS HR (detectable ctDNA pre-ICI therapy)	2.08 (1.61, 2.69)	[33]
	OS HR (detectable ctDNA during ICI therapy)	4.57 (3.03, 6.91)	[33]
	PFS HR (detectable ctDNA during ICI therapy)	3.79 (2.13, 6.75)	[33]

**Table 3:** Comparison of Liquid Biopsy (ctDNA) and Traditional Tissue Biopsy.

Characteristic	Liquid Biopsy (ctDNA)	Traditional Tissue Biopsy
Invasiveness	Minimally/Non-invasive (simple blood draw)	Invasive (surgical procedure)
Sample Accessibility	Highly accessible (even for deep-seated or widespread metastases)	Limited (may be inaccessible or high surgical risk)
Real-time Monitoring	High (repeatable for dynamic assessment)	Low (single snapshot)
Tumor Heterogeneity	Captures systemic/temporal heterogeneity from various sites	Limited (localized snapshot, may miss heterogeneity)
Turnaround Time	Faster (typically within 10 days)	Slower (can take weeks)
Risks to Patient	Minimal (e.g., bruising from blood draw)	Higher (e.g., bleeding, infection, pain, complications)
Primary Use	Treatment monitoring, MRD detection, resistance identification, early detection	Initial diagnosis, histological characterization, staging

**Table 4:** Major Challenges and Strategies for Clinical Implementation of Liquid Biopsy.

Challenge Category	Specific Issue	Proposed Strategies/Ongoing Efforts
Sensitivity/Specificity	- Low ctDNA levels in early cancer	- Technological advancements (e.g., ultra-sensitive assays like PacBio Onso, multi-omics integration)
	- False positives (Clonal Hematopoiesis)	- Improved bioinformatics for CH filtering
	- Variable assay performance	
Standardization/Reproducibility	- Lack of uniform protocols (pre-analytical, analytical, post-analytical)	- Collaborative development of universal guidelines
	- Inter-laboratory variability	- Implementation of external quality assurance programs and proficiency testing
Cost/Reimbursement	- High technology cost	- Health economic assessments to support cost-effectiveness
	- Inconsistent payer coverage	- Policy advocacy for reimbursement
	- Limited accessibility in resource-constrained settings	- “Coverage with Evidence Development” models
Clinical Evidence Gap	- Lack of RCTs demonstrating clinical benefit	- Investment in prospective RCTs targeting clinical endpoints (OS, DFS)
	- Low negative predictive value (NPV) in some settings	- Emphasis on interventional trials using MRD-guided decision-making
Interpretation Complexity	- Unclear tissue of origin for ctDNA	- Use of AI for data integration and biomarker validation
	- Ambiguity in clinical actionability of early signals	- Development of clinical pathways for positive results
	- Differentiating true progression from pseudoprogression	- Integrated diagnostics

## Data Availability

The original contributions presented in this study are included in the article. Further inquiries can be directed to the corresponding author.

## Abbreviations

The following abbreviations are used in this manuscript:

ctDNA	Circulating tumor DNA
CTCs	Circulating tumor cells
EVs	Extracellular vesicles
mtDNA	Mitochondrial DNA
MRD	Minimal residual disease
VAFs	Variant allele frequencies
CH	Clonal hematopoiesis
SNVs	Single-Nucleotide Variants
CNVs	Copy Number Variations
NGS	Next-generation sequencing
dPCR	Digital PCR
cdPCR	Chamber-based PCR
qPCR	Quantitative PCR
AI	Artificial Intelligence
ML	Machine Learning
NSCLC	Non-small cell lung cancer
HCC	Hepatocellular carcinoma
CRC	Colorectal cancer
PDAC	Pancreatic ductal adenocarcinoma
MCED	Multi-Cancer Early Detection
COPD	Chronic obstructive pulmonary disease
CAGR	Compound annual growth rate
PFS	Progression-free survival
OS	Overall survival
HR	Hazard Ratio
NPV	Negative predictive value
PPV	Positive predictive value
CEA	Carcinoembryonic antigen
CA 19-9	Carbohydrate antigen 19-9
TOO	Tissue of origin
EGFR	Epidermal growth factor receptor
ALK	Anaplastic lymphoma kinase
EML4-ALK	Echinoderm microtubule-associated protein-like 4–ALK fusion
ICB	Immune checkpoint blockade
ICI	Immune checkpoint inhibitor
CVA	Cerebrovascular accident
DVT	Deep vein thrombosis
PE	Pulmonary embolism
RCT	Randomized controlled trials
NHI	National Health Insurance (Taiwan)
